# Nucleotide-level Convolutional Neural Networks for Pre-miRNA Classification

**DOI:** 10.1038/s41598-018-36946-4

**Published:** 2019-01-24

**Authors:** Xueming Zheng, Shungao Xu, Ying Zhang, Xinxiang Huang

**Affiliations:** Department of Biochemistry and Molecular Biology, School of Medicine, Jiangsu University, Zhenjiang, China

## Abstract

Due to the biogenesis difference, miRNAs can be divided into canonical microRNAs and mirtrons. Compared to canonical microRNAs, mirtrons are less conserved and hard to be identified. Except stringent annotations based on experiments, many in silico computational methods have be developed to classify miRNAs. Although several machine learning classifiers delivered high classification performance, all the predictors depended heavily on the selection of calculated features. Here, we introduced nucleotide-level convolutional neural networks (CNNs) for pre-miRNAs classification. By using “one-hot” encoding and padding, pre-miRNAs were converted into matrixes with the same shape. The convolution and max-pooling operations can automatically extract features from pre-miRNAs sequences. Evaluation on test dataset showed that our models had a satisfactory performance. Our investigation showed that it was feasible to apply CNNs to extract features from biological sequences. Since there are many hyperparameters can be tuned in CNNs, we believe that the performance of nucleotide-level convolutional neural networks can be greatly improved in the future.

## Introduction

MicroRNAs (miRNAs) are a class of short (≈22 nt), non-coding RNAs which can regulate gene expression at the post-transcriptional level in various states, e.g. cancer, vascular diseases or inflammation^1–3^. The biogenesis of miRNAs starts with the transcription of miRNA genes, which forms primary miRNA hairpins (pri-miRNA). In the canonical pathway, pri-miRNAs are cleaved in the nucleus by the microprocessor complex, consisting of Drosha and DGCR8^4^, which produces pre-miRNAs with hairpin structure. Then, pre-miRNAs are transported to the cytosol by exportin-5 and are further processed into small RNAs duplexes by another RNase III enzyme Dicer^5,6^. Upon loading into an Argonaute (Ago) protein for target regulation, one strand of the duplex (the mature miRNA) is preferentially retained, while the other strand is degraded^7^.

The alternative miRNAs biogenesis pathway, the “mirtron” pathway, utilizes splicing to generate pre-miRNA hairpin bypassing the nuclear enzyme Drosha^8^. Then, those pre-miRNAs share the same processing pathway with the canonical miRNAs. Mirtrons come from the intronic regions of protein-coding genes, which can form short hairpins structure^9^. According to the sequence and structure, mirtrons can also be divided to canonical, 3′-tailed and 5′-tailed mirtrons. Compared to the canonical mirtrons, the 3′ or the 5′ end of those non-canonical mirtrons is also trimmed by RNA exosome after splicing^10^.

Although there are about 100,000 candidate hairpins in human genome^11^, so far less than 2,000 pre-miRNAs were reported and we were confident in only a sub-collection according to miRBase (http://wwwmirbase.org/)^12^. In those pre-miRNAs, most are annotated to be canonical. Owing to next-generation sequencing technology, many small RNA sequencing projects were done and a large quantity of sequencing data was deposit in the databases. Researchers can retrieve and analyze those data to discover new miRNAs. The analysis pipelines should following stringent criteria and the discovery need to be validated in future biological experiments^13,14^.

So far, there is a lot of computation methods developed to predict miRNAs based on diverse methodologies. Most methods are based on machine learning algorithms such as support vector machines (SVM), random forest (RF), decision tree (DT) and so on^15–17^. All of those methods are based on the exacted features of miRNAs and the performance depends heavily on selected features used in each classifier. Among these features, the length and base composition in difference sub-region of pre-miRNAs or mature mi-RNAs are often used. Most features are based on the secondary structure which is predicted by RNAfold or other softwares^18^.

Convolutional neural networks (CNNs), originally invented for computer vision, can automatically extract features by filters/kernels^19^. CNNs have already proven to be been successful for image classification and many natural language processing (NLP) tasks^20,21^. In this work we introduced a new method based on CNNs to classify miRNAs. The only information we used in our CNNs models is the pre-miRNAs sequences of human canonical miRNAs and mirtrons. Using “one-hot” encoding^22^, each nucleotide/base is casted to a four-dimensional vector. So, the pre-miRNAs can be treated as the sequences of such vectors. The greatest advantage of our method is that there is no need to select features which is heavily depended on the domain knowledge of miRNAs. Our nucleotide-level convolutional neural networks models automatically extracted features and were successfully trained on the training dataset, which showed a good performance on the test dataset. This project gives an instance of applying CNN to deal with biological sequences.

## Methods

### Training and test datasets

Human pre-miRNAs dataset (Supplementary Table S1) was retrieved from miRBase (Release 21, 06/14). According to the stringent mirtrons/canonical miRNAs annotation provided by Wen *et al*.^13^, the dataset contained 216 mirtrons and 707 canonical miRNAs. Another dataset used in this study is the putative mirtrons dataset (Supplementary Table S2) consisted of 201 novel mirtrons identified by Wen *et al*.^13^. The dataset of our supervised machine learning project was a mergence of the two datasets. Altogether, our dataset contained 1124 pre-miRNAs with imbalanced number of canonical miRNAs (707) and mirtrons (417). This was also exactly the same dataset used by Rorbach *et al*. in their recent investigation^23^. Next, we separated the dataset randomly into training (292 mirtrons/495 canonicaland pre-miRNAs) and test (125 mirtrons/212 canonical pre-miRNAs) datasets. For consistency, we partitioned the training and test datasets with the same proportion of canonical miRNAs and mirtrons. The nucleotide-level convolutional neural networks were trained on the training dataset and evaluated on the test dataset after training.

### Pre-trained one-hot encoding

Due to the different lengths of all the pre-miRNAs, we padded each pre-miRNA with different number of “N” in the end to the final maximum length of 164 (padding).

**Figure 1:**
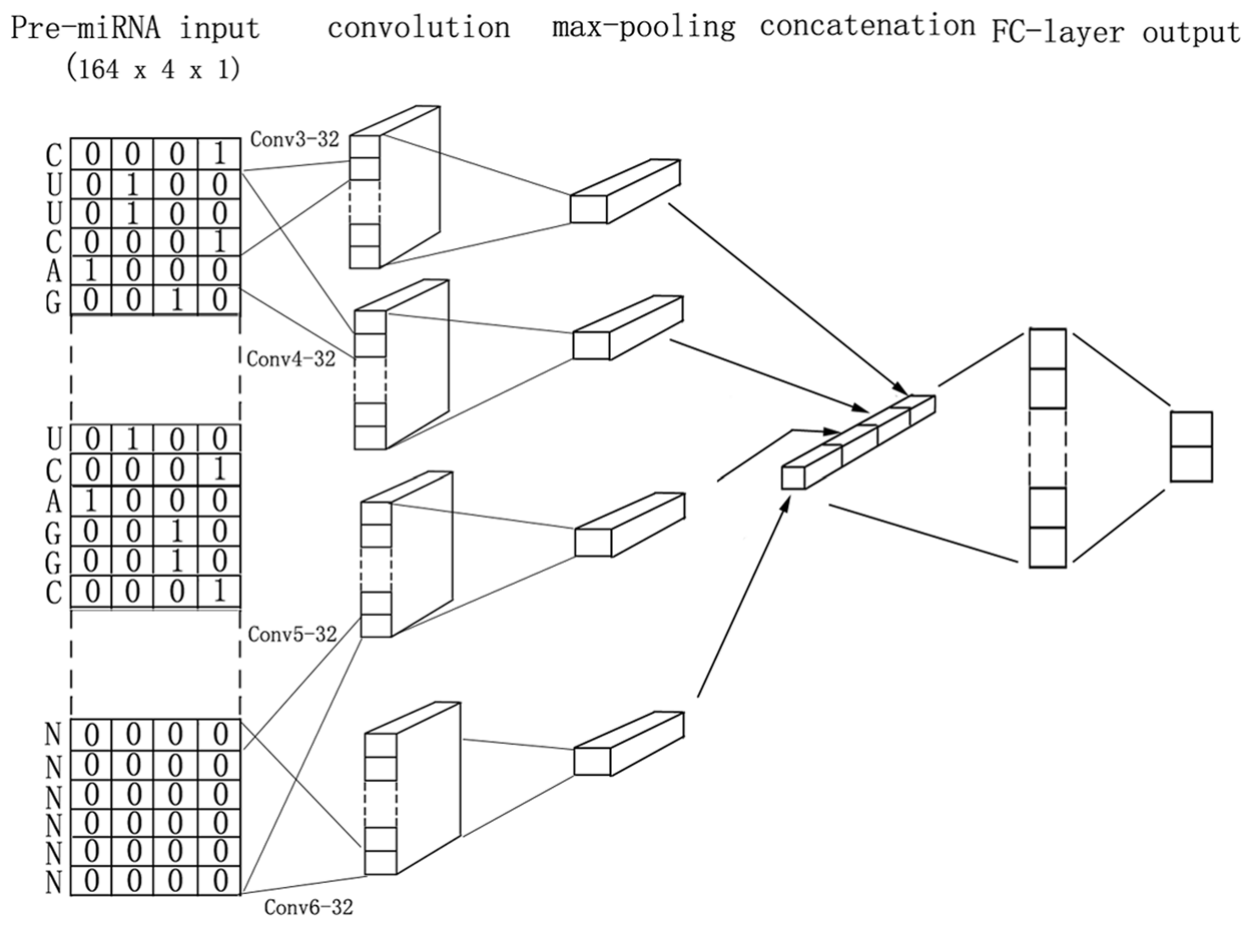
Illustration of the CNN-concat-filters model architecture for base-level pre-miRNAs classification. In CNN-concat-filters model, four kinds of filters, each of which has 32 filters, with the same width and different lengths (3, 4, 5 and 6) are employed (Conv3-32, Conv4-32, Conv5-32 and Conv6-32). In the convolution layer, each filter performs convolution on the sequence matrix and generates a feature map. The max-pooling operation then takes the largest number of each feature map. All the features are concatenated to form a 128-long feature vector for the penultimate fully-connected layer. The final layer is the softmax output which gives the probability of each classification. The shapes of the tensors as indicated in parentheses are given by height × width × channels.

Next, we encoded each base in the sequences of pre-miRNAs with “one-hot” encoding (“A”:[1, 0, 0, 0], “T/U”:[0, 1, 0, 0], “G”:[0, 0, 1, 0], “C”:[0, 0, 0, 1], “N”:[0, 0, 0, 0]). The zero padding (“N”:[0, 0, 0, 0]) have no impact on training and keeps the pre-miRNA sequences in the same length, which is essential for batch learning. Since each base was converted into a four-dimensional vector, each pre-miRNA sequence was vectorized into a vector with a dimension of (164,4) (Fig. 1).

### CNNs model architectures

We designed different CNNs architectures with one-layer of convolution and max-pooling operations. All the models have a similar architecture except the different sizes of filters used in each model. Also, we designed a mixed model (CNN-concat-filters) to study whether multiple filters can improve the performance. The architecture of the mixed model was showed in Fig. 1. First, we adopted convolution operations with different filters (filter_height = 3, 4, 5, and 6, filter_width = 4, in_channels = 1, out_channels = 32, strides: 1, padding: valid, activation: relu) to extract features from pre-miRNAs sequences. Then, the max-pooling operations^24^ took the maximum value of a particular size (164 length) as the feature corresponding to each filter. Next, all the extracted features were concatenated for the next fully-connected layer. For regularization, we employed dropout on the first fully-connected layer by a certain probability during the training process^25^. The last is the softmax layer whose output is the probability distribution over labels. In the one-kernel models, the only difference is that only one kind of filter (with the number of 128) was used in the convolution layers.

Although convolution operations can dramatically reduce the number of parameters, there are more than 19,000 parameters in each model because of the fully-connected layers. For illustration, the detailed parameters of the mixed model were showed in Table 1.

**Table 1:** The parameter and output size of each layer in the mixed model.

Layer (type)	Output Shape	Param #	Connected to
input_1 (InputLayer)	(None, 164, 4, 1)	0	
Conv3_32 (Conv2D)	(None, 162, 1, 32)	416	input_1[0][0]
Conv4_32 (Conv2D)	(None, 161, 1, 32)	544	input_1[0][0]
Conv5_32 (Conv2D)	(None, 160, 4, 32)	672	input_1[0][0]
Conv6_32 (Conv2D)	(None, 159, 4, 32)	800	input_1[0][0]
max_pooling2d_1 (MaxPooling2D)	(None, 1,1, 32)	0	Conv3_32 [0][0]
max_pooling2d_2 (MaxPooling2D)	(None, 1,1, 32)	0	Conv4_32 [0][0]
max_pooling2d_3 (MaxPooling2D)	(None, 1, 1, 32)	0	Conv5_32 [0][0]
max_pooling2d_4 (MaxPooling2D)	(None, 1, 1, 32)	0	Conv6_32 [0][0]
concatenate_1 (Concatenate)	(None, 1, 1, 128)	0	max_pooling2d_1[0][0]
			max_pooling2d_2[0][0]
			max_pooling2d_3[0][0]
			max_pooling2d_4[0][0]
flatten_1 (Flatten)	(None, 512)	0	concatenate_1[0][0]
dense_1 (Dense)	(None, 128)	16512	flatten_1[0][0]
dropout_1 (Dropout)	(None, 128)	0	dense_1[0][0]
dense_2 (Dense)	(None, 2)	258	dropout_1[0][0]

### Optimization

The loss function is defined as the cross entropy between the predicted distribution over labels and the actual classification^26^.

1 $${\rm{C}}{\rm{r}}{\rm{o}}{\rm{s}}{\rm{s}} \mbox{-} {\rm{e}}{\rm{n}}{\rm{t}}{\rm{r}}{\rm{o}}{\rm{p}}{\rm{y}}=-{\sum }_{i=1}^{{\rm{n}}}{{\rm{y}}}_{{\rm{i}}}{{\rm{l}}{\rm{o}}{\rm{g}}{\rm{s}}}_{{\rm{i}}}$$

(n: the number of labels, y_i_: the actual probability for label i, s_i_: predicted probability for label i). The goal of our machine learning is to minimize the mean loss function and find the right weights and biases. The model was trained on the training dataset using back-propagation to update gradients on the parameters^27^.

### Method evaluation

The performances of our CNN classifiers were measured on the test dataset. We calculated the following performance measures.

(TP: true positive, TN: true negative, FP: false positive, FN: false negative)

Sensitivity (Recall) shows the true positive rate:

2 $${\rm{Sensitivity}}={\rm{TP}}/(\mathrm{TP}+\mathrm{FN})$$

Specificity shows the true negative rate:

3 $${\rm{Specificity}}={\rm{TN}}/(\mathrm{TN}+\mathrm{FP})$$

F1-Score is the harmonic mean of precision and sensitivity:

4 $${{\rm{F1}}}_{{\rm{score}}}={\rm{2}}\ast {\rm{TP}}/(2\ast {\rm{TP}}+{\rm{FP}}+\mathrm{FN})$$

Matthews Correlation Coefficient (MCC) is in essence a correlation coefficient between the observed and predicted binary classifications.

5 $${\rm{MCC}}=({\rm{TP}}\ast {\rm{TN}}-{\rm{FP}}\ast {\rm{FN}})/{[(\mathrm{TP}+\mathrm{FP})\ast (\mathrm{TP}+\mathrm{FN})\ast (\mathrm{TN}+\mathrm{FP})\ast (\mathrm{TN}+\mathrm{FN})]}^{1/2}$$

Accuracy shows the overall correctness of prediction:

6 $${\rm{Accuracy}}=(\mathrm{TP}+{\rm{TN}})/({\rm{TP}}+{\rm{TN}}+{\rm{FP}}+{\rm{FN}})$$

## Results

Since the convolutional neural networks can automatically extract features from images and sentences, we wonder whether CNNs can be used to predict the classification of pre-miRNAs. Here, we used “one-hot” encoding for pre-RNA vectorization and five kinds of model architectures were designed. Different from traditional machine learning methods, our methods only used the raw sequences, instead of selected features, of pre-miRNAs.

The parameter and the size of output tensor in each layer are showed. The flow of tensors in computation map of the model is also indicated.

**Figure 2:**
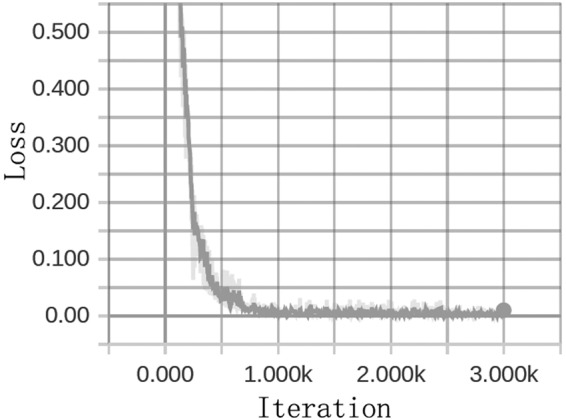
The loss graph during training. The loss was defined as the cross entropy between predicted value and the actual one. With the iteration of training, the loss dramatically decreases and finally tends to zero. The loss graph is from the CNN-concat-filters model. Horizontal axis: iteration times. Vertical axis: loss value.

Each model was successfully trained on the training dataset. The loss graphs showed that our nucleotide-level convolutional neural networks models learned very fast (Fig. 2). But with the iteration of training, the prediction accuracy of test dataset remains the same although the loss of training dataset continuously decreases, indicating overfitting. Hence, we stopped the training process of the models with the generalization error (difference between the losses of train and test) which can not be avoided. All the training process was finished in less than 20 minutes in an ordinary laptop computer (i5 CPU, 4 G RAM).

Finally, we evaluated the performances of our models on the test dataset and compared them with traditional machine learning methods. The results showed that the prediction accuracies of all our models were about 90% and the specificities were more than 94%, while sensitivities were less than 90% (Table 2). The considerably lower sensitivity for mirtrons than for canonical miRNAs is probably due to the small number of mirtrons in the dataset. We also assessed our classifier performances with F1 score and correlation MCC. It seems that CNN-filter6–128 model has the best performance and using multiple sizes of filters (CNN-concat-filters model) can not promote the performance of the model. Compared to other machine learning methods, our nucleotide-level convolutional neural networks models have comparatively higher specificity, high F1 value and lower sensitivity for mirtrons prediction^23^.

**Table 2:** Performances comparison of our models with traditional machine learning methods. Our models were trained on the training dataset and evaluated on the test dataset. Our models were compared with traditional machine learning methods. The performance data of the traditional machine learning methods were from Rorbach, G., *et al*.^23^. “—” means “data not provided in the original paper”.

Model	Sensitivity	Specificity	F1	MCC	Accuracy
CNN-filter3-128	0.846	0.945	0.890	0.795	0.895
CNN-filter4-128	0.786	0.980	0.871	0.781	0.883
CNN-filter5-128	0.861	0.955	0.903	0.819	0.908
CNN-filter6-128	0.871	**0.970**	**0.916**	0.845	0.920
CNN-concat-filters	0.846	0.975	0.904	0.827	0.910
Support Vector Machines	0.926	0.945	0.901	0.859	—
Random Forest	0.870	0.957	0.883	0.836	—
Linear Discriminant Analysis	0.935	0.919	0.881	0.830	—
Logistic Regression	0.875	0.941	0.867	0.816	—
Decision Tree	0.861	0.943	0.863	0.808	—
Naive Bayes	0.875	0.894	0.824	0.746	—

## Discussion

This work is our preliminary investigation on miRNA classification using convolutional neural networks. The results showed that CNNs successfully extracted features from RNA sequences and the accuracies of our predictors reached and even exceeded 90%. But, all our models showed relatively higher specificity and lower sensitivity for mirtrons, which means considerable mirtrons were misclassified into canonical pre-miRNAs. This phenomenon may be caused by the imbalanced numbers of pre-miRNAs and mirtrons in the dataset.

As we know, the architecture of the CNNs is vital important to the performance of the CNN-based classifier. In this work, we tried several different sizes of filters and one max-pooling strategy. Our experiments indicated that filter selection may help to improve the performance and the usage of different sizes of filters resulted in an average performance. Since we only used one-layer CNN in our models, more sophisticated architectures with multiple convolution layers may lead to improved performances. Furthermore, there are many hyperparameters that can be tune to a specific classifier, there is great possibility to optimize our models in the future investigation.

There is also import information in mature miRNAs, which is used to extract features in other traditional machine learning methods. Since we only use the pre-miRNAs sequences for classification, the model performance may be greatly improved if the mature miRNAs sequences can be used. Moreover, we only use “one-hot” encoding to convert the pre-miRNAs sequences, other nucleotide/base embedding methods should be investigated in the future.

## Conclusion

In this work, we proposed nucleotide-level convolutional neural networks models to predict the classification of human pre-miRNAs. Using “one-hot” encoding and base padding, all the pre-miRNAs were converted into matrixes with the same size. We employed one-layer convolution and max-pooling operations with different sizes of filters followed by two fully connected layers. Compared with other machine learning methods, which is heavily dependent on hand-extracted features, our methods can automatically extract features by convolution and max-pooling operations. Since the only information we need is the labeled sequences of pre-miRNAs, our nucleotide-level convolutional neural networks methods are easy to implement.

Our results showed that all the models were successfully trained on the training dataset and had a good performance on the test dataset. Our work indicated that convolutional neural networks can be used for biological sequence classification.

## Supplementary information

Dataset 1

Dataset 2

## Data Availability

The source code is freely available through GitHub (https://github.com/zhengxueming/cnnMirtronPred), distributed under the version 2 of the general public license (GPLv.2).
